# Supplementary material for: Examining the bacterial diversity including extracellular vesicles in air and soil: implications for human health
Source: PLoS One. 2025 Apr 1;20(4):e0320916. doi: 10.1371/journal.pone.0320916 (PMC11960916; doi:10.1371/journal.pone.0320916)
Supplement: S1 Fig — (PPTX) [file pone.0320916.s001.pptx]

## Slide 1
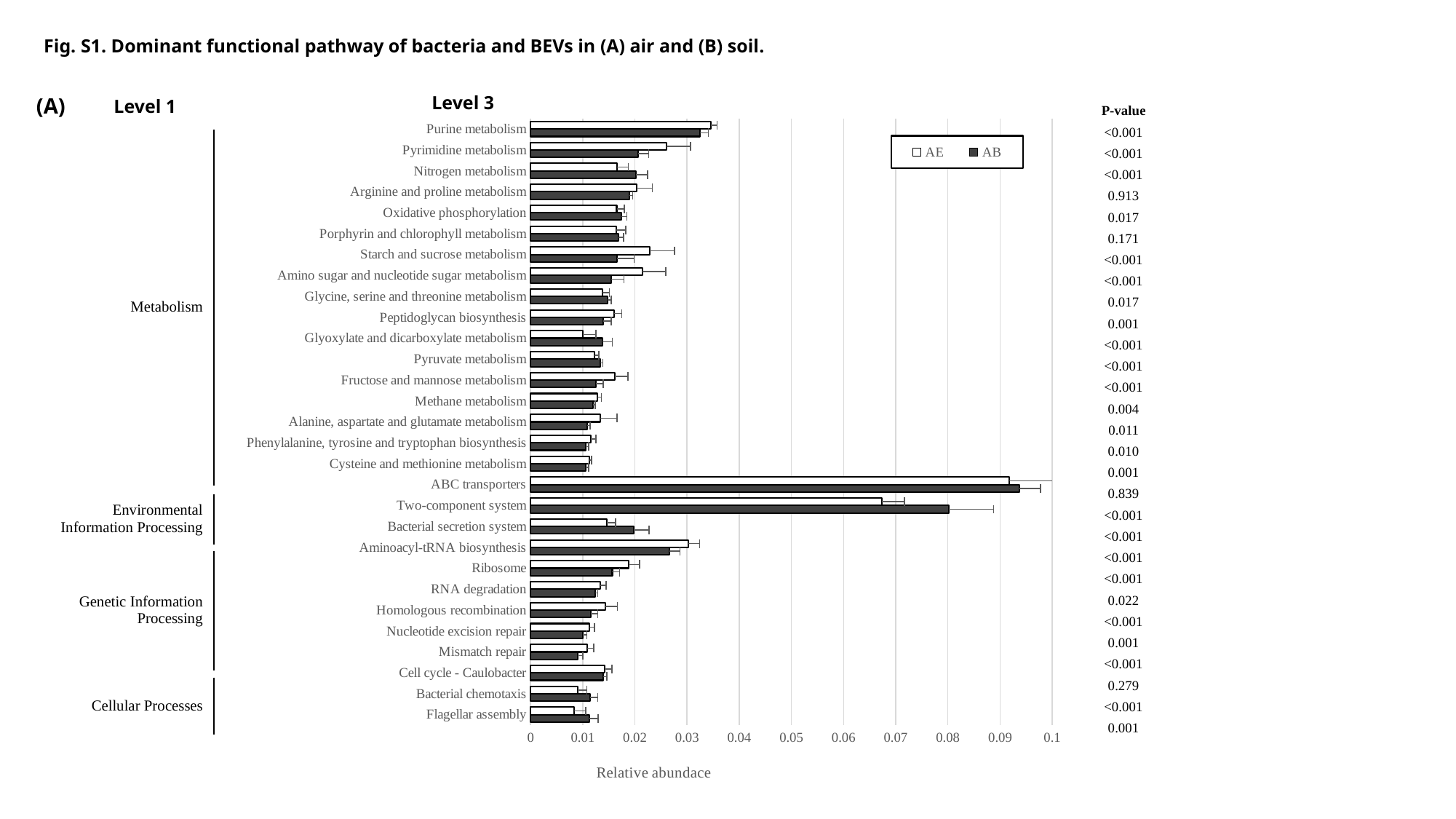

Fig. S1. Dominant functional pathway of bacteria and BEVs in (A) air and (B) soil.
Level 3
(A)
Level 1
| P-value |
| --- |
| <0.001 |
| <0.001 |
| <0.001 |
| 0.913 |
| 0.017 |
| 0.171 |
| <0.001 |
| <0.001 |
| 0.017 |
| 0.001 |
| <0.001 |
| <0.001 |
| <0.001 |
| 0.004 |
| 0.011 |
| 0.010 |
| 0.001 |
| 0.839 |
| <0.001 |
| <0.001 |
| <0.001 |
| <0.001 |
| 0.022 |
| <0.001 |
| 0.001 |
| <0.001 |
| 0.279 |
| <0.001 |
| 0.001 |
### Chart
| Category | AB | AE |
|---|---|---|
| Flagellar assembly | 0.01123867877830428 | 0.008380705985496274 |
| Bacterial chemotaxis | 0.0114036374405052 | 0.009070216724145356 |
| Cell cycle - Caulobacter | 0.013910926080455367 | 0.014248734358358316 |
| Mismatch repair | 0.009053693175174087 | 0.01086014770929531 |
| Nucleotide excision repair | 0.010059264311063198 | 0.01122313041285814 |
| Homologous recombination | 0.011548604831317212 | 0.014348681417328266 |
| RNA degradation | 0.012372506778580716 | 0.013313119359799239 |
| Ribosome | 0.015661907128323202 | 0.018795893227978037 |
| Aminoacyl-tRNA biosynthesis | 0.026621980966038825 | 0.030229268994756666 |
| Bacterial secretion system | 0.019843313021840323 | 0.014608200846903008 |
| Two-component system | 0.08019878752574772 | 0.06732594601228388 |
| ABC transporters | 0.09376928604483274 | 0.09182105132082694 |
| Cysteine and methionine metabolism | 0.010541223486783656 | 0.011284046959953949 |
| Phenylalanine, tyrosine and tryptophan biosynthesis | 0.010594800792949019 | 0.011518963610471075 |
| Alanine, aspartate and glutamate metabolism | 0.010886620942400385 | 0.013413545950650458 |
| Methane metabolism | 0.01194896184102788 | 0.012747514778676645 |
| Fructose and mannose metabolism | 0.012478421383975206 | 0.01614253905802908 |
| Pyruvate metabolism | 0.01331520974905055 | 0.012252497562257299 |
| Glyoxylate and dicarboxylate metabolism | 0.0137460703444809 | 0.009995726515283299 |
| Peptidoglycan biosynthesis | 0.013963961985171 | 0.016026270031493914 |
| Glycine, serine and threonine metabolism | 0.01470444873186966 | 0.013809469443278384 |
| Amino sugar and nucleotide sugar metabolism | 0.015434467673036797 | 0.021434026930671347 |
| Starch and sucrose metabolism | 0.016569923924511436 | 0.022871279750023808 |
| Porphyrin and chlorophyll metabolism | 0.016907718373734643 | 0.016388931121865 |
| Oxidative phosphorylation | 0.017405818385425847 | 0.016506734236853834 |
| Arginine and proline metabolism | 0.01888227179768107 | 0.02036781165102024 |
| Nitrogen metabolism | 0.020244549469728344 | 0.016590983215709027 |
| Pyrimidine metabolism | 0.020666016163077148 | 0.02606074149960974 |
| Purine metabolism | 0.032535954016993364 | 0.03460200001709573 || Metabolism |
| --- |
| Environmental Information Processing |
| --- |
| Genetic Information Processing |
| --- |
| Cellular Processes |
| --- |

## Slide 2
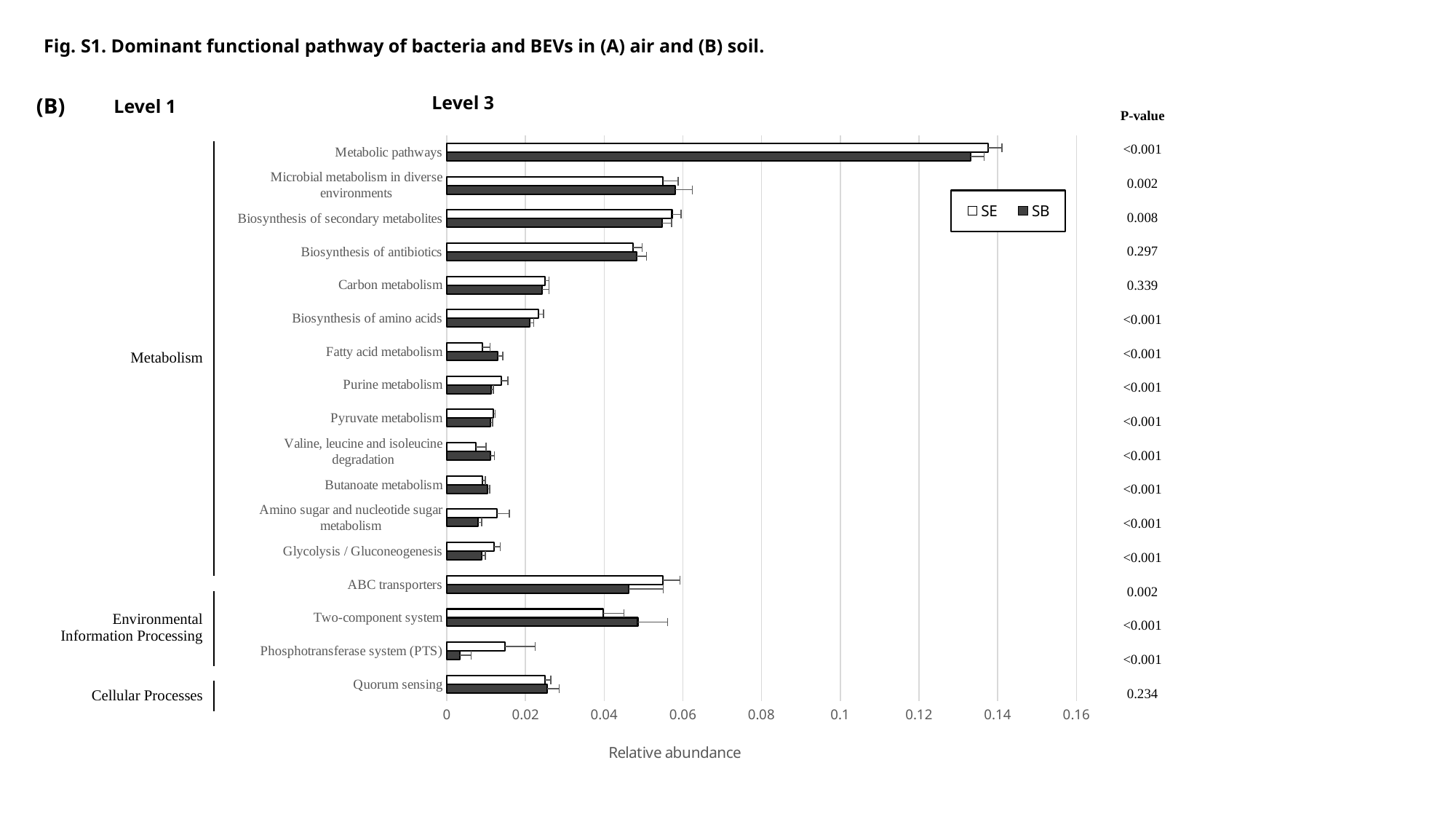

Fig. S1. Dominant functional pathway of bacteria and BEVs in (A) air and (B) soil.
Level 3
(B)
Level 1
| P-value |
| --- |
| <0.001 |
| 0.002 |
| 0.008 |
| 0.297 |
| 0.339 |
| <0.001 |
| <0.001 |
| <0.001 |
| <0.001 |
| <0.001 |
| <0.001 |
| <0.001 |
| <0.001 |
| 0.002 |
| <0.001 |
| <0.001 |
| 0.234 |
### Chart
| Category | SB | SE |
|---|---|---|
| Quorum sensing | 0.025523935886752364 | 0.02497281916698688 |
| Phosphotransferase system (PTS) | 0.003324952879237119 | 0.014839625810820241 |
| Two-component system | 0.048547636338371426 | 0.039715668573027725 |
| ABC transporters | 0.04619949836320693 | 0.05501374540084809 |
| Glycolysis / Gluconeogenesis | 0.00891013841187721 | 0.012005808084069642 |
| Amino sugar and nucleotide sugar metabolism | 0.007972852679176634 | 0.012763099333839846 |
| Butanoate metabolism | 0.010287701425854043 | 0.009000319567502305 |
| Valine, leucine and isoleucine degradation | 0.011028483901565312 | 0.007377393680320431 |
| Pyruvate metabolism | 0.011113565202935206 | 0.011892479953757107 |
| Purine metabolism | 0.01121779844899912 | 0.013864863313785409 |
| Fatty acid metabolism | 0.012960185120724969 | 0.009029681406749288 |
| Biosynthesis of amino acids | 0.02109021752462614 | 0.023270466805792256 |
| Carbon metabolism | 0.024299164059852208 | 0.02492324161756354 |
| Biosynthesis of antibiotics | 0.0482507938438376 | 0.0473575730739329 |
| Biosynthesis of secondary metabolites | 0.05470182444204492 | 0.05724375704895088 |
| Microbial metabolism in diverse environments | 0.058032661039346185 | 0.054903818026165736 |
| Metabolic pathways | 0.13319186322968726 | 0.13765646108834406 || Metabolism |
| --- |
| Environmental Information Processing |
| --- |
| Cellular Processes |
| --- |
