# Supplementary material for: Examining the bacterial diversity including extracellular vesicles in air and soil: implications for human health
Source: PLoS One. 2025 Apr 1;20(4):e0320916. doi: 10.1371/journal.pone.0320916 (PMC11960916; doi:10.1371/journal.pone.0320916)
Supplement: S2 Fig — (PPTX) [file pone.0320916.s002.pptx]

## Slide 1
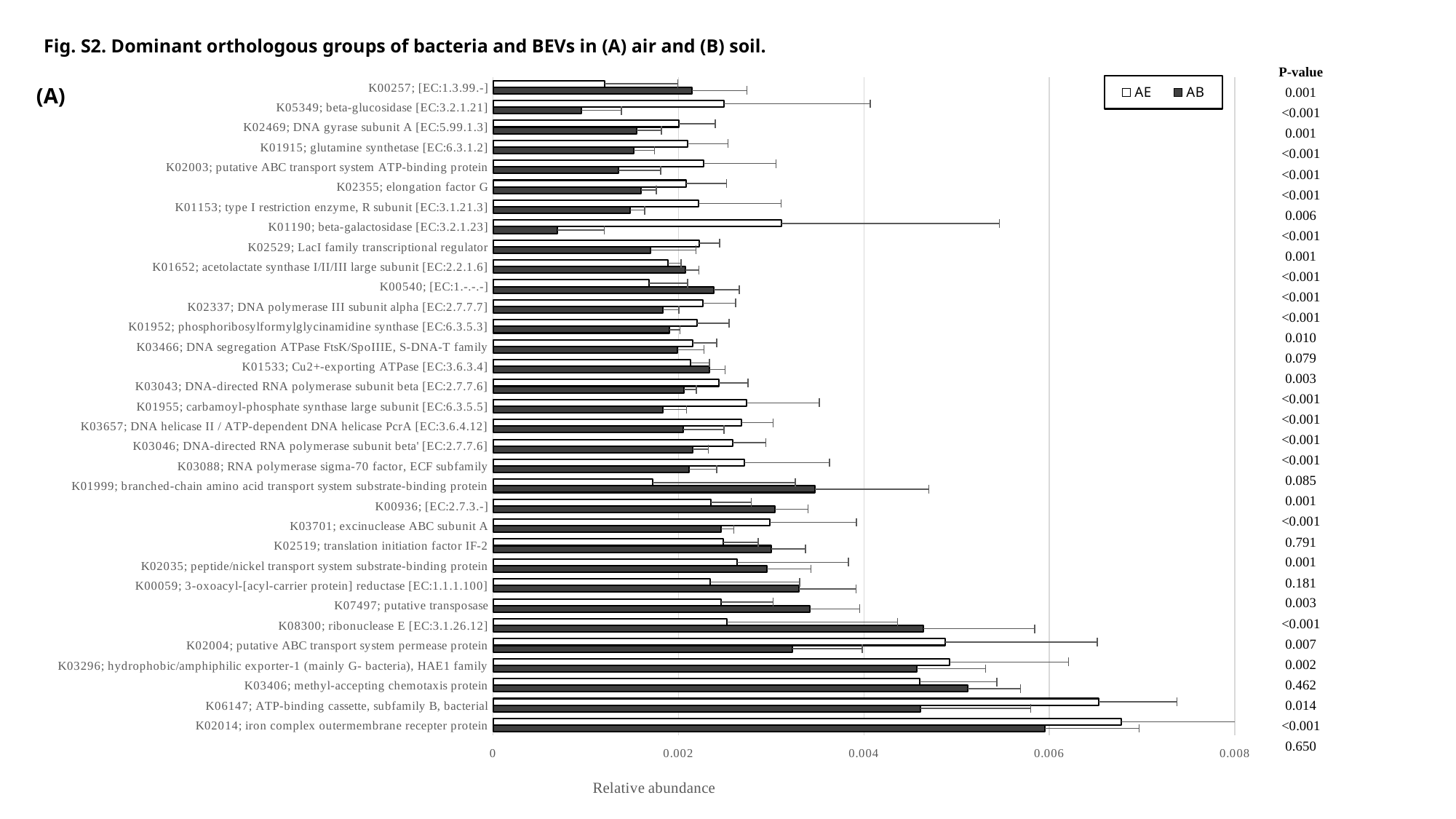

Fig. S2. Dominant orthologous groups of bacteria and BEVs in (A) air and (B) soil.
### Chart
| Category | AB | AE |
|---|---|---|
| K02014; iron complex outermembrane recepter protein | 0.005954035434944764 | 0.00677622723122928 |
| K06147; ATP-binding cassette, subfamily B, bacterial | 0.004606944957846462 | 0.006534250428509443 |
| K03406; methyl-accepting chemotaxis protein | 0.0051198222364343925 | 0.00460451164300271 |
| K03296; hydrophobic/amphiphilic exporter-1 (mainly G- bacteria), HAE1 family | 0.004571721657610115 | 0.004926429049825355 |
| K02004; putative ABC transport system permease protein | 0.0032304599469556647 | 0.004875730165596753 |
| K08300; ribonuclease E [EC:3.1.26.12] | 0.004638246899860447 | 0.0025236101166520833 |
| K07497; putative transposase | 0.003414829068672918 | 0.0024574859496629274 |
| K00059; 3-oxoacyl-[acyl-carrier protein] reductase [EC:1.1.1.100] | 0.003299361135350231 | 0.0023430611573102217 |
| K02035; peptide/nickel transport system substrate-binding protein | 0.0029512397623560806 | 0.0026316809635676414 |
| K02519; translation initiation factor IF-2 | 0.0030025191263698594 | 0.002481843339394761 |
| K03701; excinuclease ABC subunit A | 0.00245770532386252 | 0.0029846393349277434 |
| K00936; [EC:2.7.3.-] | 0.0030370347587083506 | 0.002348545719710349 |
| K01999; branched-chain amino acid transport system substrate-binding protein | 0.003474765621357319 | 0.0017247237112442094 |
| K03088; RNA polymerase sigma-70 factor, ECF subfamily | 0.0021161755633634606 | 0.0027107924273679085 |
| K03046; DNA-directed RNA polymerase subunit beta' [EC:2.7.7.6] | 0.002151276804256757 | 0.0025816333645389505 |
| K03657; DNA helicase II / ATP-dependent DNA helicase PcrA [EC:3.6.4.12] | 0.00205316025066177 | 0.0026769798236066255 |
| K01955; carbamoyl-phosphate synthase large subunit [EC:6.3.5.5] | 0.001831180413221717 | 0.002735690523843294 |
| K03043; DNA-directed RNA polymerase subunit beta [EC:2.7.7.6] | 0.002058354968089459 | 0.002437314429753493 |
| K01533; Cu2+-exporting ATPase [EC:3.6.3.4] | 0.0023338806197684293 | 0.0021305418772088 |
| K03466; DNA segregation ATPase FtsK/SpoIIIE, S-DNA-T family | 0.0019883044318341403 | 0.0021513026168666887 |
| K01952; phosphoribosylformylglycinamidine synthase [EC:6.3.5.3] | 0.0018990064335801535 | 0.0021995206964569593 |
| K02337; DNA polymerase III subunit alpha [EC:2.7.7.7] | 0.0018340676278961357 | 0.0022632152705534 |
| K00540; [EC:1.-.-.-] | 0.0023807285000048734 | 0.001682047985527948 |
| K01652; acetolactate synthase I/II/III large subunit [EC:2.2.1.6] | 0.0020786674412912685 | 0.0018890384902215307 |
| K02529; LacI family transcriptional regulator | 0.0017003989003981257 | 0.0022233408804170837 |
| K01190; beta-galactosidase [EC:3.2.1.23] | 0.0006912291760604441 | 0.003108815366194109 |
| K01153; type I restriction enzyme, R subunit [EC:3.1.21.3] | 0.0014751272776260043 | 0.0022158497142602206 |
| K02355; elongation factor G | 0.0015973449379266822 | 0.0020830286272540085 |
| K02003; putative ABC transport system ATP-binding protein | 0.0013555567375599408 | 0.002273927497057517 |
| K01915; glutamine synthetase [EC:6.3.1.2] | 0.0015197724364881687 | 0.002097453250542697 |
| K02469; DNA gyrase subunit A [EC:5.99.1.3] | 0.0015469072568025345 | 0.0020051044166732486 |
| K05349; beta-glucosidase [EC:3.2.1.21] | 0.0009493844918380921 | 0.002492970354049724 |
| K00257; [EC:1.3.99.-] | 0.0021451403812839907 | 0.0012042881418001525 || P-value |
| --- |
| 0.001 |
| <0.001 |
| 0.001 |
| <0.001 |
| <0.001 |
| <0.001 |
| 0.006 |
| <0.001 |
| 0.001 |
| <0.001 |
| <0.001 |
| <0.001 |
| 0.010 |
| 0.079 |
| 0.003 |
| <0.001 |
| <0.001 |
| <0.001 |
| <0.001 |
| 0.085 |
| 0.001 |
| <0.001 |
| 0.791 |
| 0.001 |
| 0.181 |
| 0.003 |
| <0.001 |
| 0.007 |
| 0.002 |
| 0.462 |
| 0.014 |
| <0.001 |
| 0.650 |
(A)

## Slide 2
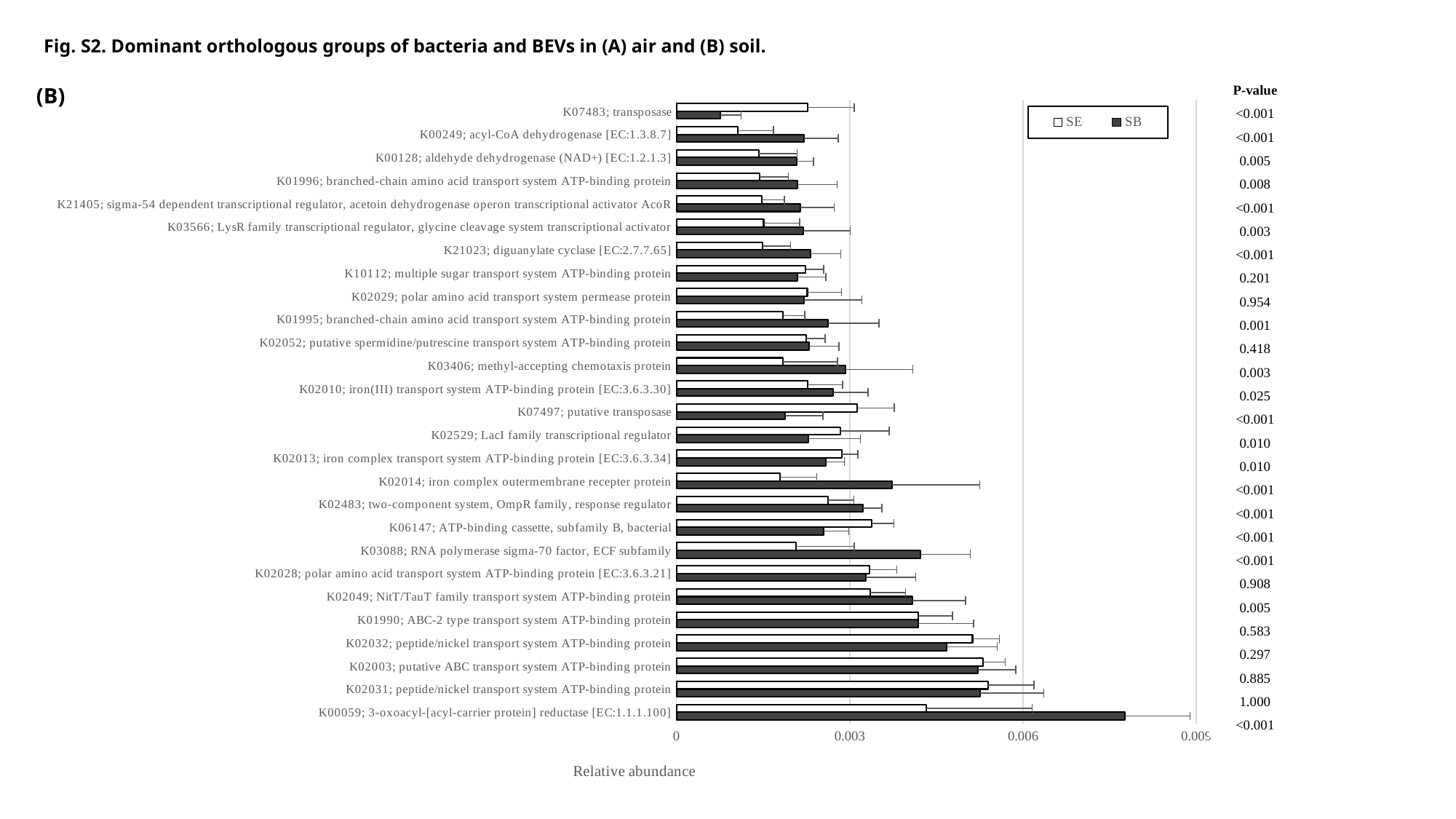

Fig. S2. Dominant orthologous groups of bacteria and BEVs in (A) air and (B) soil.
(B)
| P-value |
| --- |
| <0.001 |
| <0.001 |
| 0.005 |
| 0.008 |
| <0.001 |
| 0.003 |
| <0.001 |
| 0.201 |
| 0.954 |
| 0.001 |
| 0.418 |
| 0.003 |
| 0.025 |
| <0.001 |
| 0.010 |
| 0.010 |
| <0.001 |
| <0.001 |
| <0.001 |
| <0.001 |
| 0.908 |
| 0.005 |
| 0.583 |
| 0.297 |
| 0.885 |
| 1.000 |
| <0.001 |
### Chart
| Category | SB | SE |
|---|---|---|
| K00059; 3-oxoacyl-[acyl-carrier protein] reductase [EC:1.1.1.100] | 0.007767991121772227 | 0.004327429143044417 |
| K02031; peptide/nickel transport system ATP-binding protein | 0.005260000671796516 | 0.005391120114881035 |
| K02003; putative ABC transport system ATP-binding protein | 0.005218692778729686 | 0.005303910867106674 |
| K02032; peptide/nickel transport system ATP-binding protein | 0.004677073255432288 | 0.0051244981873529594 |
| K01990; ABC-2 type transport system ATP-binding protein | 0.004186397466831841 | 0.0041882661925450185 |
| K02049; NitT/TauT family transport system ATP-binding protein | 0.004083215060989452 | 0.003357371563854444 |
| K02028; polar amino acid transport system ATP-binding protein [EC:3.6.3.21] | 0.0032825105902550907 | 0.0033481886750687025 |
| K03088; RNA polymerase sigma-70 factor, ECF subfamily | 0.004226576172683185 | 0.00207264076234332 |
| K06147; ATP-binding cassette, subfamily B, bacterial | 0.0025524680615249787 | 0.0033784637568253207 |
| K02483; two-component system, OmpR family, response regulator | 0.0032327196071996562 | 0.002630742884520632 |
| K02014; iron complex outermembrane recepter protein | 0.0037280960198507822 | 0.001793708360533822 |
| K02013; iron complex transport system ATP-binding protein [EC:3.6.3.34] | 0.0025843654956501996 | 0.0028607138164645747 |
| K02529; LacI family transcriptional regulator | 0.002286265327024318 | 0.002839011737841487 |
| K07497; putative transposase | 0.0018823724412891224 | 0.0031310169743638803 |
| K02010; iron(III) transport system ATP-binding protein [EC:3.6.3.30] | 0.002714829603657677 | 0.0022723123638866195 |
| K03406; methyl-accepting chemotaxis protein | 0.0029287657519984165 | 0.0018435946633205366 |
| K02052; putative spermidine/putrescine transport system ATP-binding protein | 0.0023028589892441927 | 0.0022493327337795277 |
| K01995; branched-chain amino acid transport system ATP-binding protein | 0.002625990806920607 | 0.0018444712069219612 |
| K02029; polar amino acid transport system permease protein | 0.002204332007342089 | 0.0022653172075959072 |
| K10112; multiple sugar transport system ATP-binding protein | 0.0020984429594211027 | 0.002234906978973935 |
| K21023; diguanylate cyclase [EC:2.7.7.65] | 0.002325268172159653 | 0.0014873658068940785 |
| K03566; LysR family transcriptional regulator, glycine cleavage system transcriptional activator | 0.0022007426575166257 | 0.0015105019244909357 |
| K21405; sigma-54 dependent transcriptional regulator, acetoin dehydrogenase operon transcriptional activator AcoR | 0.0021410540071422103 | 0.0014782962212539726 |
| K01996; branched-chain amino acid transport system ATP-binding protein | 0.0020934650522670857 | 0.001441927326363627 |
| K00128; aldehyde dehydrogenase (NAD+) [EC:1.2.1.3] | 0.002078729930691135 | 0.0014340478247035272 |
| K00249; acyl-CoA dehydrogenase [EC:1.3.8.7] | 0.0022084531358387612 | 0.0010655635099711645 |
| K07483; transposase | 0.0007655912715093933 | 0.0022717493433161485 |
